# ORIENTATE: automated machine learning classifiers for oral health prediction and research

**DOI:** 10.1186/s12903-023-03112-w

**Published:** 2023-06-20

**Authors:** Inmaculada Gomez-Rios, Esteban Egea-Lopez, Antonio José Ortiz Ruiz

**Affiliations:** 1 Department of Dermatology, Stomatology, Radiology and Physical Medicine, Universidad de Murcia, Murcia, Spain; 2 Dept. Information Technologies and Communications, Universidad Politecnica de Cartagena (UPCT), Cartagena, Spain

**Keywords:** Machine learning, Classification, Special health care needs, Deep sedation, Predictive dentistry, Second sedation risk

## Abstract

**Background:**

The application of data-driven methods is expected to play an increasingly important role in healthcare. However, a lack of personnel with the necessary skills to develop these models and interpret its output is preventing a wider adoption of these methods. To address this gap, we introduce and describe ORIENTATE, a software for automated application of machine learning classification algorithms by clinical practitioners lacking specific technical skills. ORIENTATE allows the selection of features and the target variable, then automatically generates a number of classification models and cross-validates them, finding the best model and evaluating it. It also implements a custom feature selection algorithm for systematic searches of the best combination of predictors for a given target variable. Finally, it outputs a comprehensive report with graphs that facilitates the explanation of the classification model results, using global interpretation methods, and an interface for the prediction of new input samples. Feature relevance and interaction plots provided by ORIENTATE allow to use it for statistical inference, which can replace and/or complement classical statistical studies.

**Results:**

Its application to a dataset with healthy and special health care needs (SHCN) children, treated under deep sedation, was discussed as case study. On the example dataset, despite its small size, the feature selection algorithm found a set of features able to predict the need for a second sedation with a f1 score of 0.83 and a ROC (AUC) of 0.92. Eight predictive factors for both populations were found and ordered by the relevance assigned to them by the model. A discussion of how to derive inferences from the relevance and interaction plots and a comparison with a classical study is also provided.

**Conclusions:**

ORIENTATE automatically finds suitable features and generates accurate classifiers which can be used in preventive tasks. In addition, researchers without specific skills on data methods can use it for the application of machine learning classification and as a complement to classical studies for inferential analysis of features. In the case study, a high prediction accuracy for a second sedation in SHCN children was achieved. The analysis of the relevance of the features showed that the number of teeth with pulpar treatments at the first sedation is a predictive factor for a second sedation.

## Background

The application of data-driven methods is expected to play an increasingly important role in healthcare [1, 2], as they can be a particularly effective tool for diagnosis (disease presence) or prognosis (risk of future outcome), among other tasks. To mention a few representative examples: they have been used for the identification and classifications of different types of cancers [3], or to predict individualized optimal drug doses for patients [4]; they have also been used for natural language processing of medical records for improving the accuracy of appendicitis diagnoses [4] and to prevent hypoxaemia during surgery [5]. They also allow to analyze how reliable medical information is conveyed on social networks [6]. Machine Learning (ML) is the branch of artificial intelligence that encompasses methods to make computers learn how to do some task from experience (data). In particular, ML uses statistical methods to predict outcomes in future data [2], that is, to make classifications or predictions. ML algorithms are *trained* with data and fall into two broad categories: supervised learning, which uses data that have been previously labeled (with the desired or correct value), and unsupervised learning, which uses data that have not been labeled. As described in recent works [1, 2] the prediction accuracy of ML systems in some fields may meet or surpass that of the experts. Finally, ML systems handle well large volumes of high dimensional data [2] and, therefore, can be a very effective tool for large scale preventive healthcare programs, for tasks such as large-scale screening [7]. As an example, *AutoPrognosis*, a software that automatically builds an ensemble of predictive ML models, has been used to predict cardiovascular diseases: in a study done over 5 years with a sample of 4801 cases it was able to predict correctly 368 more cases than alternative classical methods [8].

However, there are a number of challenges that prevent the wider adoption of these methods, both technical, such as the need for appropriate and interoperable data models [2] and high-quality data [7]; and operational, such as their integration in the clinical workflow [2, 7]. One particular problem pointed out by Callahan [2] is the *lack of personnel with the necessary skills to develop these models and interpret their output*. Developing an ML model for healthcare is a demanding task, which includes problem selection, data curation, development, and validation [7]. Even though the application of sophisticated ML algorithms is relatively easy thanks to high-level programming libraries such as *scikit-learn* [9], clinical practitioners usually lack the technical background required for its use. And conversely, technicians often lack the required clinical skills for appropriate model evaluation and refinement, which usually results in a time-consuming exchange of requirements and model prototypes between the two groups [10]. In summary, a first step before deploying ML systems appropriate for preventive healthcare is the development of a validated model for the problem at hand, which is complicated due to a lack of complementary skills of the involved actors.

To address this problem, in this paper we introduce and describe ORIENTATE (applicatiOn of machine leaRning for classIfication of dENTal pATiEnts), a software that allows the use of sophisticated ML algorithms for the classification and prediction of oral health conditions by clinical practitioners lacking specific technical skills (*users*). Given a collected *dataset*, the application basically provides a web-based interface for the selection of its features (*predictors*) that may be useful in the prediction of some other variable of interest (*target*). It then generates a number of ML classification models and cross-validate them against subsets of a training set, selecting the best model according to some performance metric, and evaluating it against a validation set. The tool shows a detailed summary of the evaluation with graphs that facilitates the explanation of the ML model results, using global interpretation methods [11], and an interface for the prediction of new input samples. The previous procedure summarizes a first stage of use of the tool, where researchers generate and evaluate a suitable prediction model from a given dataset. The integration of the tool into the clinical workflow would come in a second stage, where the validated model can be used, either with the provided interface or a custom (not developed yet) application. The practitioner would generate a prediction, just by filling in the corresponding predictors for a new patient, that may guide her in the planning of further treatments, complementing the information on caries risk and lesion management provided by clinical tools such as the Caries Management by Risk Assessment (CAMBRA) [12] or the International Caries Classification and Management System (ICCMS) [13].

A first goal of the tool is to facilitate testing different combinations of predictors according to the user criteria. It, therefore, allows an exploratory analysis that can be used to validate hypotheses about the influence of different features on the target variable [7]. The tool guides the user in the development process of the model, offering the selection of the class of interest, alternatives for imputation of missing data and selection of the performance metric. It is also agnostic with respect to the dataset used, that is, it can be used with different datasets as long as they follow a minimal shared data model. Once the exploratory phase has finished, the obtained model has usually enough quality (prediction performance) as to be used in production. Therefore, trained models can be used in preventive tasks by clinical practitioners without technical training. A second goal is to perform systematic searches for the best combination of predictors for a target variable. A custom feature selection algorithm has been implemented, which tests thousands of combinations of predictors and returns the best model found together with an extensive explanatory report of the feature relevance and interactions. Finally, the explanatory report can be used to perform an inferential analysis of the data, complementary to classical statistical studies [14]. This analysis can be done by examining the relevance and interactions of the predictors used by the model, an information that is visually provided by the generated report. Users with varying technical expertise may benefit from the use of ORIENTATE. Researchers only need to complement their dataset with an additional metadata CSV file in a simple format and then proceed with the model generation. In fact, providing metadata forces the researcher to analyze her data and collect and organized it in a systematic way, facilitating the discovery of errors and removing redundant information. The generated models can be critically analyzed by other researchers or practitioners just by examining the provided visual reports. The validation of the best model found is internally done by the tool by evaluating a separated test set from the available data. The prediction results for all the samples are provided to the user, so that she may assess directly the quality of the predictions, even individually, even with plots of the relevance of each predictor for the final result. Finally, as mentioned previously, clinical practitioners can generate new predictions just by filling out a form and then examining the provided report.

To illustrate the use of the application and the performance of the various functionalities, we use a particular case study: a dataset that describes the oral health condition in two populations, one with healthy children and another one with SHCN, was collected to compare the treatments performed under deep sedation in both populations. The dataset was analyzed with classical statistical methods in a previous work [15, 16]. This particular dataset is described in detail in the “Material and methods of the previous study” section. We apply to this dataset the feature selection algorithm implemented by ORIENTATE and show how it finds a set of predictors that results in an accurate predictor model of the need for a second sedation. Next, we examine the relevance of the predictors and their interactions and discuss how they agree with the inferences derived in our previous work using classical statistical methods.

ORIENTATE source code is freely available in our repository, as well as the datasets used in the case study and an additional public dataset, whose format has been adapted, from [17], which can be used to test the application.

## Implementation

ORIENTATE has been implemented as a web-based application and so it is independent of the platform and only requires a web browser to be used.

### Data source

The application is independent of the dataset used, as long as it is provided in the format described in the Machine learning workflow section. The particular dataset used in our use case is described in detail in Material and methods of the previous study section.

### Libraries

A first prototype of the web application was implemented in Python v3.9.12 with Flask v2.0.3. Machine learning algorithms are implemented with SciKit-Learn v1.1.1 and SHAP v.0.41.0.

### Functionality

**Fig. 1:**
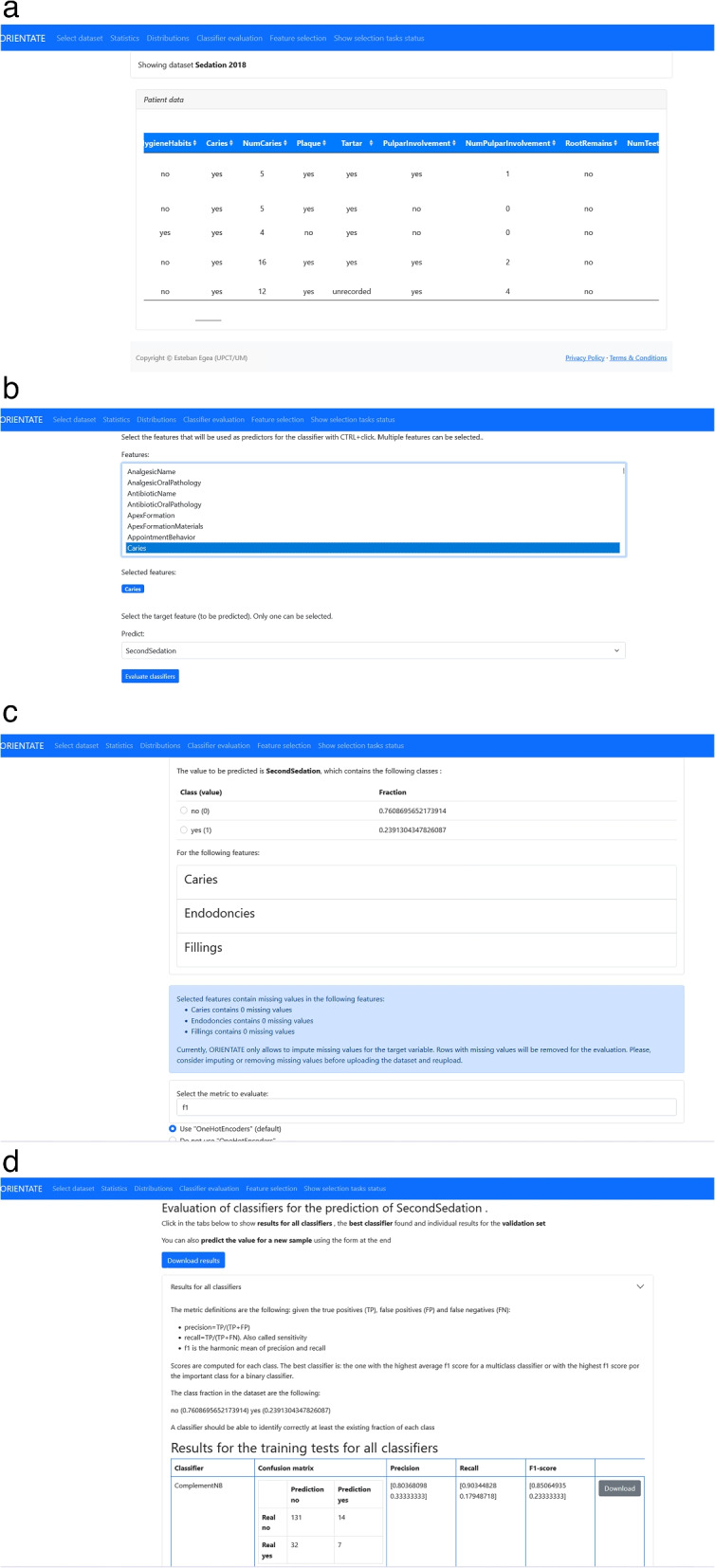
Screenshots of the web application interface and steps for the evaluation of classifiers

ORIENTATE allows uploading of the working dataset which is stored for future use. Once the dataset has been uploaded or selected from the available ones, the entry point of the web application shows a view of the current dataset and a menu with the available actions, as shown in Fig. 1a. The menu entries with Statistics and Distributions provide a statistical summary of the dataset and the empirical distributions of the variables respectively. The main functionality can be found in Classifier evaluation, (CE) and Feature selection (FS) as described next.

**Fig. 2:**
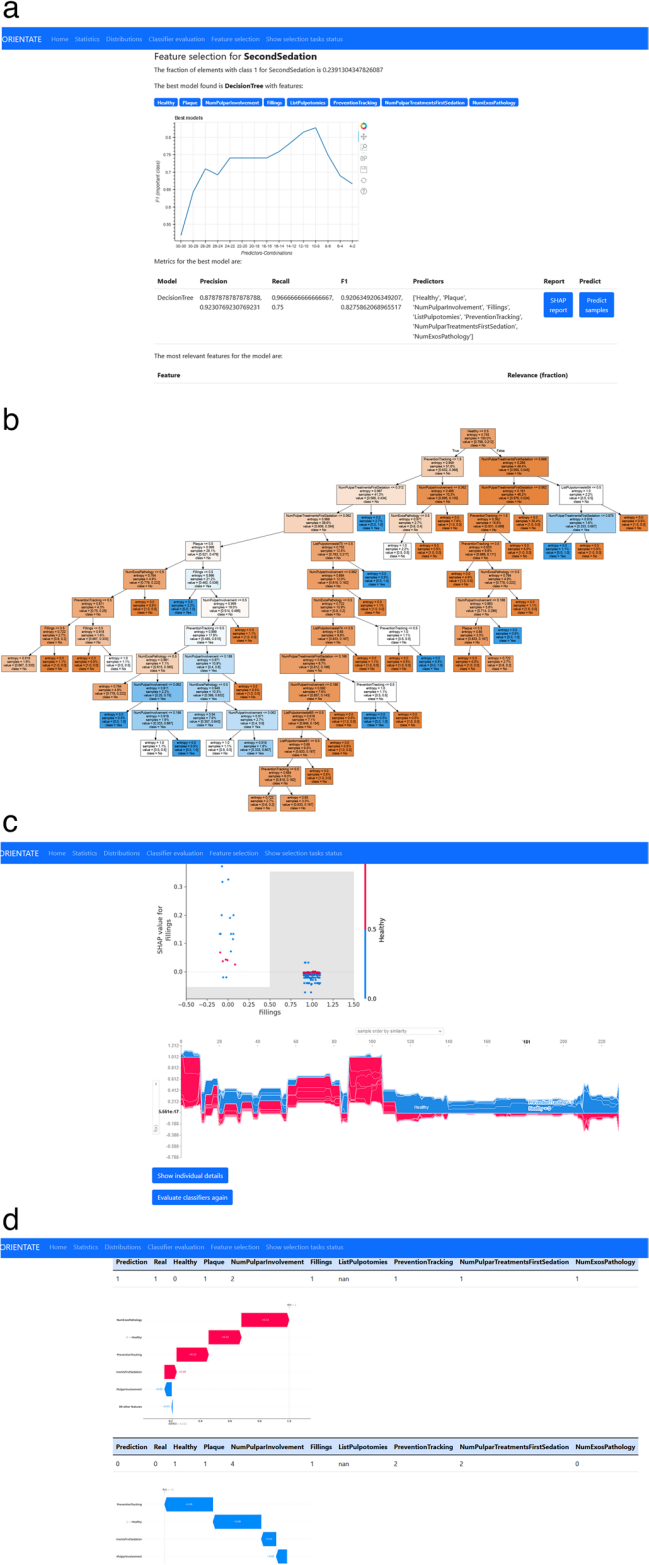
Screenshots of the web application interface for the selection of features

**Fig. 3:**
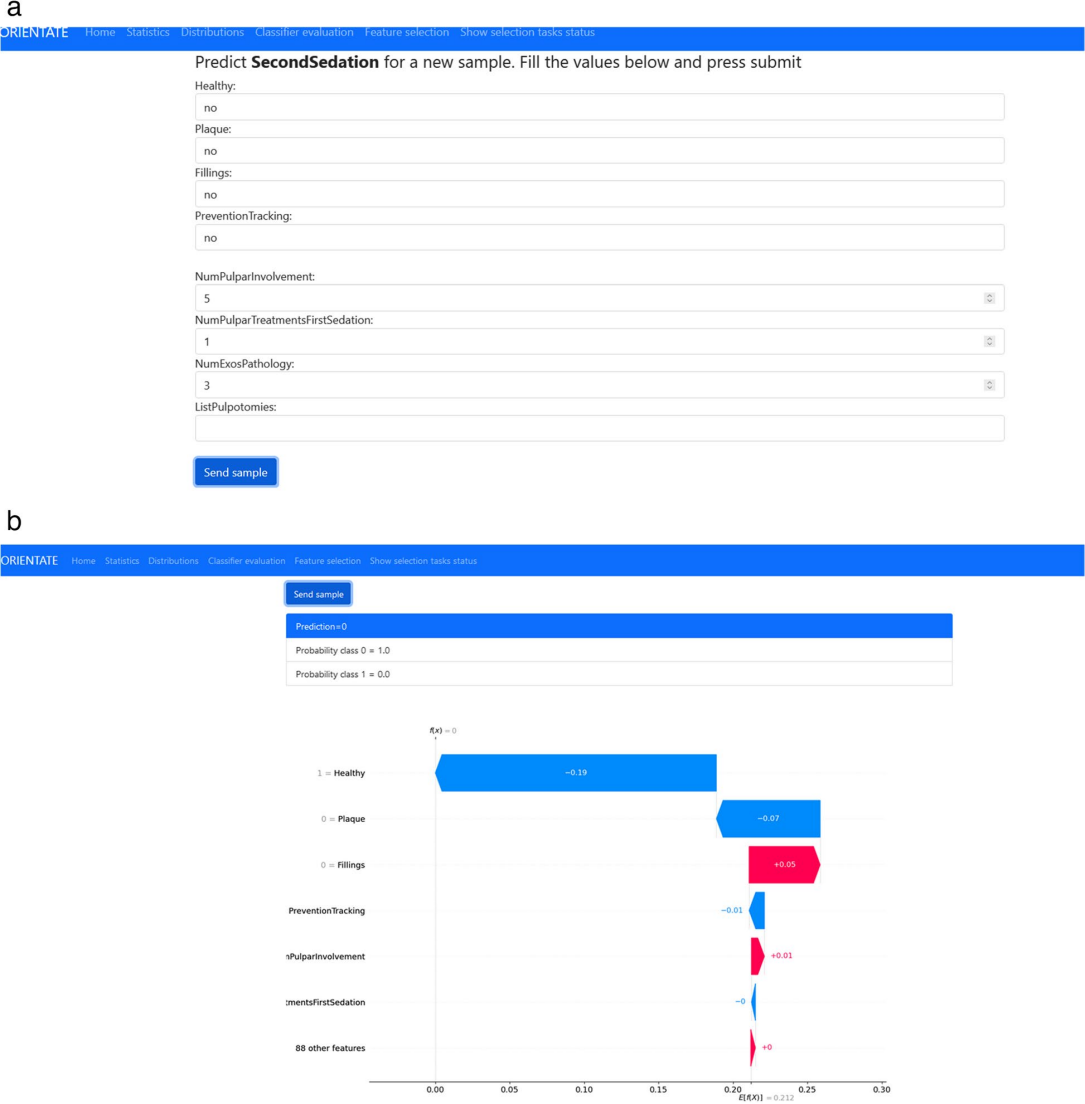
Screenshots of the web application interface for the prediction of new samples

- *Classifier evaluation (CE)*. This link performs a search for the best classifier to predict a chosen target feature, given a set of features selected by the user. The application collects the information from the user in two separate steps as shown in Fig. 1b and c. First, the user selects the target variable to be predicted and the set of features to be used as predictors. Next, the user selects the class of interest for binary variables. For instance, for the variable *Caries* the user selects whether she is interested in presence (class 1) or absence (class 0) of caries. For multiclass variables this action is skipped. The application shows the available options, and, in case of missing values (usually not registered) in the target feature, the user selects an imputation method for the missing values. Currently, the user can only select between removal of rows with missing values or replacement with a constant value. Once the input is collected, the application searches for the best classifier among a set of predefined types of classifiers, which are described in detail in the “Machine learning workflow” section, and shows a summary of the results. This step is described in detail in the following section. A partial view of the evaluation results window is shown in Fig. 1d. Our goal is to simplify the interpretation of the results to clinical practitioners, so in this step an explanation of the performance metrics is provided and additional information is presented to the user. The summary includes the metric scores and confusion matrix obtained for all the classifiers tested [18]. The best classifier found according to the metric is separately highlighted. In addition, the predictions of the best classifier for each sample in the validation test are also displayed. That is, for all the samples in the validation test, the application shows a table with a row with the predicted value, the real value, the probability for each class and the values of the predictors. Finally, the user can predict, with the best classifier found, the value of a new sample by inserting the values of the predictors in a form provided. All the results can be downloaded for further use. The goal of this functionality is to let the user find a good classifier when the set of predictors (features) is previously established, for instance, by the expert knowledge of the clinical practitioner. The results allow to examine the differences between classifiers. It also allows to test how minor variations in the feature set, such as the replacement of a few predictors, influence the classifier performance.
- *Feature selection (FS)*. In many cases, however, the clinical practitioner is interested in finding out which features may help to predict a certain target variable. In fact, the goal of many studies is to uncover and describe the associational relationships between a set of features and the target variable. Similarly, our tool allows to search for the set of features that result in more accurate classifiers. This functionality can be viewed as an extension of CE, because the above procedure is repeated for different subsets of the feature set. Since it is usually not possible to test all the combinations of features, the application uses a feature selection algorithm that is described in detail in the Machine learning workflow section. The application shows the user the same steps as in CE: first, a feature selection window, where the user selects the set of features for the search, and then the class selection and imputation window. In this case, the application informs the user of the number of combinations to be tested and asks for a name for the selection task to be created. Since this task is time-consuming usually, a background task is executed and the user is directed to another tab where the progress of the pending tasks is shown. Once the background task has finished the search for the best combination of features, the user can view the results. First, the user is presented with a summary of the feature selection search which includes: 1) a plot of the best metric achieved for each of the combinations of features tested, as shown in Fig. 2a; 2) the best model and features found; 3) the relative relevance of each of the features for the best model found; and 4) when the best model found is a DecisionTree classifier, the application additionally shows the actual decision tree generated by the model, shown in Fig. 2b. Our goal again is to provide the information in a way that can be interpreted by the clinical user as easily as possible. Relative feature relevance helps the user understand the importance that the model assigns to each feature, which can be critically assessed by the user. The generated decision tree is particularly helpful in this sense, since it allows the user to actually reproduce the decision process of the model and assess its soundness. Moreover, users can use the tree to derive their own protocols, adapting it to the clinical practice by refining or simplifyingthe tree. When clicking on the SHAP report button for the found model, further support for interpretation is provided. In this case, a SHAP report of the model is generated. SHAP [11] is a method to explain individual predictions and generate global interpretations. The application computes the SHAP values and generates a number of global interpretation plots, which include feature importance, feature dependence, interactions and clustering of feature relevance, as shown in the snapshots in Fig. 2c. Finally, the user can inspect individual results. In that case, the application displays the individual predictions and corresponding SHAP explanations for all the elements in the dataset. The snapshot in Fig. 2d shows the particular values of the features for that individual as well as the predicted and real values of the target variable. Below, a SHAP waterfall plot displays how the values of the features contribute to the particular decision. In the Case study section we use our case study to discuss and describe in detail those plots.
- *Predictions for new samples*. Once a satisfactory classifier has been found, either by CE or FE, it can be used to predict the result for new samples. ORIENTATE generates automatically a form, shown in Fig. 3a, for the user to fill the values of the predictors for a new sample. After submitting, the results are displayed to the user, as shown in Fig. 3b, including the probabilities for the different predicted classes and a plot for the SHAP values of the new samples to interpret how the feature values have influenced the newly predicted sampled.

### Machine learning workflow

In this section we describe the ML procedures used by the tool. Most of them are implemented as functions that are combined to yield the desired result. For instance, Feature Selection uses data selection and preprocessing and then calls repeatedly classifier evaluation with different combinations of features. Details can be checked in the source code in our repository.

- *Data format*. Data are compiled into a CSV file, with an additional metadata file with custom format, providing a description of the type of variable of each column and its range of values and optionally a legend to facilitate showing content in a human-friendly format. Variable types may be continuous variables, bounded and unbounded discrete variables, categorical variables encoded as integers with a given valid range and *tooth-lists*. The latter maps a set of teeth to their number according to the ISO 3950:2016 standard [19], which designates teeth or areas of the oral cavity using two digits.
- *Data selection and preprocessing*. ORIENTATE allows the user to select the target and the predictor features from the set of features present in the dataset. Once they are selected, the tool analyzes the number of classes present in the target feature. For binary classification, the user is prompted to mark the relevant class, which is used later to select the best model as the one with the best selected performance metric for that class. The performance metric used can also be chosen, among precision, recall and f1-score [18]. For multiclass classification, the best model is that with the best average performance metric over all the classes. The presence of missing data in the target feature is analyzed. In case there is missing data, the user must select an imputation method for it. Since the dataset used comprised only categorical data, the options are to remove or fill with some constant value the missing data. Additional imputation methods are available (use average or more frequent value). Afterward, the predictor features are prepared for the classifiers by normalizing continuous variables and one-hot encoding tooth-lists and optionally categorical variables. A transformation pipeline with the imputation and encoding is generated and the dataset is passed for the evaluation of the classifiers.
- *Classifier evaluation*. The dataset is split into a train set with 80% of the samples and a validation set with the remaining ones. Then, a default set of classifiers is evaluated with 3-fold cross-validation on the training set and a series of performance scores is generated for each classifier. The default set of classifiers includes single instance classifiers: logistic regression, support vector machines with linear and Gaussian RBF kernels, linear classifier with stochastic gradient descent learning, different *naive* Bayes classifiers, Gaussian Process classifier, multilayer perceptron; and ensemble methods: random forest, gradient boosting (XGBoost), AdaBoost and *stacking* with random forest, Gaussian and logistic regression. All the classifiers are initialized with a set of previously selected default parameters, not configurable at the moment by the user. Automated hyperparameter optimization might be added as an additional stage for the tool, if necessary, but we consider that the potential gains are not worthy for the current functionality and given the increased complexity of the software. Once the cross-validation is done for each of the classifiers, the tool selects as best classifier the one with the best performance metric as described before. The best classifier is trained with the complete training set and finally the validation set is predicted with the fitted model, resulting in a final set of performance scores. The results from this process are a set of metrics for each of the classifiers evaluated as well as for the fitted best model: confusion matrix, recall, precision, f1-score and ROC AUC. All of them are gathered and shown to the user by the tool. The best model is saved to be used for the prediction of new input samples. For the validation set, the prediction probabilities for all the samples in the set are also stored and shown. Since for most of the classifiers these are not precise estimates of the class probabilities, they need to be calibrated before being shown to the user.
- *Feature selection*. When the user completes the data selection and preprocessing stage described before, a set of potential features is ready. At this point two different algorithms are used, depending on the number of features in the set (*N*). The total number of combinations to test is $c=2^N - N -1$, since we include at least two features, and when this number is too high, it is not computationally feasible to test them all. So we have arbitrarily set $N=13$ as the maximum number of features for fully testing all the combinations. Obviously, this parameter should be set according to the available processing power. For instance, in our server (i9-9280X CPU, 2 NVIDIA RTX 2080Ti GPUS, 32 GB RAM), testing $N=11$ features, that is, $c=2036$ combinations, takes around one hour. Therefore, when the number of features is up to 13, $N \le 13$, a background processing task iteratively executes the classifier evaluation procedure described above for each of the combinations in the dataset, that is, first with the *N* features, then all the $N-1$ combinations and so on until testing all combinations of 2 features. The results of each evaluation are saved to the database. Otherwise, when $N > 13$, a custom feature selection algorithm is executed in the background task. Feature selection is, in general, a hard problem due to the combinatorial explosion, and there are multiple algorithms available and different methods [20]. We employ here a so-called wrapper method [20], using the predictor performance as the objective function, and implement a simple variant of a Sequential Backward Selection (SBS) algorithm. Though this kind of algorithms typically returns a local optimum, they can produce good results, are computationally feasible, and we avoid the additional complexity of more sophisticated methods, which we do not consider critical for our goals. Our algorithm proceeds as follows: at each iteration there is a set of predictors to be tested, which we call $\mathbb {T}$ with size *T*, and a removal parameter, *k*. The algorithm is initialized with $\mathbb {T}$ including the total number of features, *N*, and evaluates the performance for all the classifiers in the default set as described in the *classifier evaluation* procedure. The results and metrics obtained in the evaluation are saved to the database, including the best classifier found. Next it evaluates all combinations of $T-k$ elements from the total set and saves the metrics and best classifier found. Afterward, the predictors in the test set are replaced by the predictors used by the best classifier. Notice that now the number of elements in the test set, *T*, has been reduced by *k* elements. And again all the combinations of $T-k$ elements are evaluated as described above. This procedure is repeated until the test set contains fewer than 2 elements. Finally, we select the best classifier found from all the combinations evaluated. We illustrate the algorithm with an example, where we start with $N=20$ features, so that $\mathbb {T}$ contains the all the 20 features, and use $k=2$. After the first evaluation with all the features, we evaluate all the combinations of 18 elements, taken from the 20 elements. Then we replace the test set with the features used by the best classifier found, that is, the test set contains now 18 elements, and evaluate all the combinations of 16 elements from the 18 features in the test set. Notice how the size of the test set is reduced at each iteration, in this example by $k=2$ elements.
- *Generation of reports*. Reports are generated at the end of the classifier evaluation (CE) or feature selection (FS) procedures. For the former, the confusion matrices and metrics obtained for all the evaluated models are shown. In addition, the probabilities predicted by the best model for all the elements in the validation set are displayed. For the feature selection procedure, we show a plot of the f1-metric for the best classifier for all the evaluated combinations and the list of the predictors for the best classifier found. If the best found model is able to compute the feature importances, those are shown. Additionally, when the best found model is a DecisionTree, the application displays the generated tree. Afterwards, the SHAP values for the model are computed [11]. For this, we use the specialized TreeExplainer [21], otherwise, we use an ExactExplainer [11] with an independent masker of 1000 samples. Once the values are computed, different plots are shown to the user as described in the Functionality section, including waterfall plots for individual explanations.

## Results

We have applied our tool to a dataset collected for a previous study [15, 16]. The goal of that study was to describe the oral health condition in two populations, one with healthy children and another one with SHCN, that is, children who due to physical, medical, developmental or cognitive conditions require special consideration when receiving dental treatment [22]; and to compare the treatments performed under deep sedation in both populations. This way we illustrate two of the main goals of our tool: (1) the performance of the feature selection algorithm and prediction power of the trained classifier and (2) its capabilities as an alternative to classical statistical studies. We first briefly describe our previous work, then describe the feature selection results and the interpretation plots provided by ORIENTATE for inferences.

All these results are put in context in the Discussion section, where we discuss our results in comparison with the findings of our previous study [15, 16] and related works.

### Material and methods of the previous study

A cohort study was carried out with children who had been treated for their oral pathology under deep sedation in a private clinic in Cartagena (Murcia, Spain), during the years 2006 to 2018, both included. 274 medical records of patients aged 2 to 18 years were reviewed, including both children with an optimal general health condition which we will call "healthy children", and SHCN children [22]. Patients whose medical records did not correctly show demographic data or health status data were excluded. Finally, a total of 230 clinical records (109 (47.4%) healthy and 121 (52.6%) SHCN) were included in the study. Within the SHCN, 19.50% were general developmental disorders, 14.70% were encephalopathies and cerebral palsy, Down syndrome 3.5%, intellectual and/or motor disability 4.7%, and other syndromes 10%. The sample consisted of 142 men (61.74%) and 88 women (38.26%), with a mean age of 7.10 ± 3.40 years. The mean age of the healthy patients was 5.04 ± 2.42 years and that of the group of SHCN children was 8.95 ± 3.09 years. Most of children were 4, 6, 7, 8 and 9 years old.

The information that was collected from the clinical records was: from the first visit, sex, age, systemic health status (healthy or SHCN child), reason for the first sedation, information on the oral health status prior to the intervention (hygiene habits, plaque, tartar, caries lesions, pulp involvement, root remains and absences). On the day of the intervention, the following data were collected: the types of treatments performed (fillings, direct pulp protections, pulpotomies, pulpectomies, endodontics, apex formation, scaling, scaling and root planing, application of fluoride and extractions) and the number of teeth treated. Follow-up data include attendance or not at the check-up where the presence of plaque is assessed, the need for medication due to oral pathology, and improvement at mealtime. In addition, it was recorded the performance of the prevention follow-up behavior in appointments, classifying the patient as "cooperative" or "non-cooperative", according to whether the patient allowed the dentist and/or hygienist to carry out their work; the motivation of parents in the oral care of their children, classifying them as “motivated” or “not motivated”, according to whether they are involved in the care of their children mouth and implement at home the dietary and oral hygiene recommendations that are given to them. Finally, treatments performed subsequently without sedation, year of last check-up and tracking time. The data of the successive sedations included the cause, failed treatments, treatments performed, number of sedations and time from the first to the last sedation.

The collected data described above were incorporated into a dataset and systematically encoded and labeled according to the type of data and data format described in the Machine learning workflow section: binary variables are labeled by a descriptive name, continuous or integer variables are prefixed with *Num*, while tooth-lists are prefixed with *List*. For instance, the presence of caries is encoded with a binary variable (0 or 1) and labeled *Caries*, the number of teeth with caries is labeled *NumCaries* and the list of teeth with caries is labeled *ListCaries*. Notice that some information is redundant.

Our previous study is a *traditional* one, where the data were statistically analyzed with *classical* methods. In particular, a descriptive analysis of all the variables was performed. Continuous quantitative variables were compared two by two using a T-test, a T-test with Welch’s correction, or a Mann-Whitney test, depending on the assumptions of normality and homoscedasticity. For discrete variables, the Kruskal-Wallis test was used together with the Dwass-Steel-Critchlow-Fligner test to determine the two-to-two differences. To establish the relationship between discrete qualitative or quantitative variables, contingency tables were made with Pearson $\chi ^2$ or Fisher exact test, depending on whether or not the assumptions were fulfilled. Finally, a test of equality of proportions without continuity correction was used for testing proportions. Additional information on these methods can be found in [15].

### Feature selection performance

The dataset compiled from the previously described cohort study contains 102 different features, some of them redundant. From them, 30 potential features of interest were extracted by consensus between 2 odontologists, ruling out those not determining, such as being referred or need to take oral medication, those with insufficient number of data, such as endodontic treatments and apical formation, or those for whom there were not enough variability in the data. The *Feature Selection (FS)* functionality was executed on them, with *SecondSedation* as target variable, a binary variable that indicates whether the patient was subjected to a second sedation. Or in other words, the goal of the test is to find the classifier that is able to better predict the need for a second sedation from all or a subset of the 30 selected predictors (features), using f1-score as peformance metric. Since the number of features $N=30$ is greater than 13, our SBS algorithm was executed, taking 1 hour and 40 minutes to evaluate $c=2360$ combinations of predictors.

**Fig. 4:**
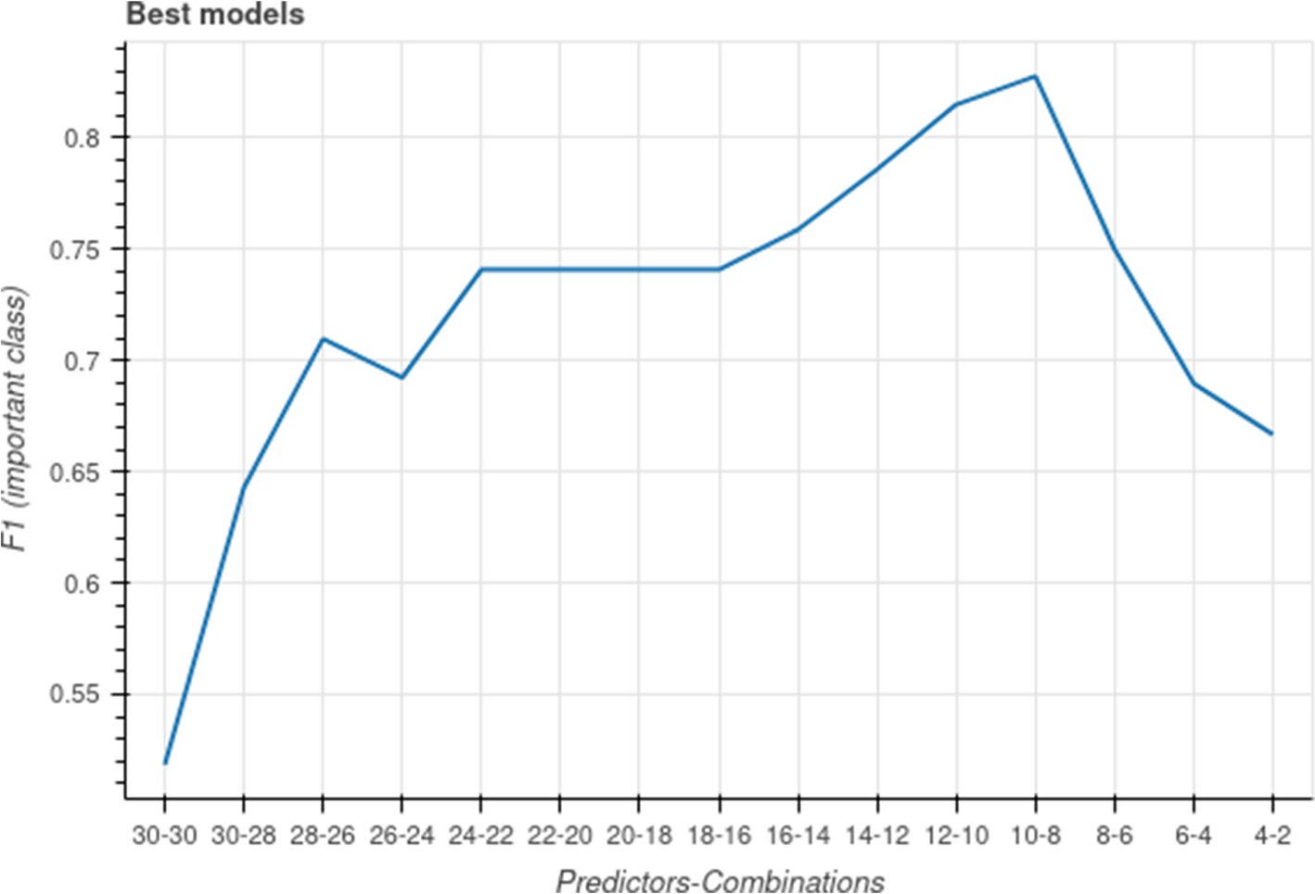
f1 score obtained by the best classifier found versus the number of features in the test set and number of combinations evaluated (Predictors-Combinations). The f1 score shown is for the class of interest, in this case, the occurrence of a second sedation

Figure 4 shows the f1-score for the best classifiers found versus the number of features in the test set and combinations evaluated. As can be seen, the best classifier is found when the feature set contains 10 features and all the combinations of 8 features were tested. Therefore, the best classifier uses 8 predictors, listed in Table 1. The best model is a DecisionTree classifier, therefore, the generated tree can be displayed and the decision process of the model can be analyzed. Actually, the generated tree has been shown previously as example in Fig. 2b. The scores achieved by the classifier on the validation set are shown in Table 2. Additionally, the ROC (AUC) achieved is 0.92. It has to be taken into account that the dataset is *imbalanced*, that is, only 24% of the patients underwent a second sedation. Therefore, it is easier to predict the absence of second sedation. Anyway, the model found is able to accurately predict the need for a second sedation, with a f1-score for the class of interest of 0.83 and a balanced accuracy of 0.81. There is an additional imbalance in the dataset, that of the healthy to SHCN patients: the number of second sedations for SHCN patients is much higher than that for healthy children. In Table 3 we compare the scores when conditioning on the two groups. As seen, only 6% of the healthy patients undergo a second sedation, and the model has difficulties to correctly predict those sedations, as expected, but for the SHCN patients it is accurate in the predictions.

**Table 1:** Features used by the best classifier found by the feature selection algorithm

Feature	Type	Description
Healthy	binary	Systemic health status
Plaque	binary	Presence of plaque before first sedation
Fillings	binary	Fillings done at first sedation
PreventionTracking	ternary	Compliance with prevention follow-ups
NumPulparInvolvement	integer	Number of teeth with pulpar involvement before first sedation
NumPulparTreatmentsFirstSedation	integer	Number of pulpar treatments done at first sedation
NumExosPathology	integer	Number of extractions due to pathology done at first sedation
ListPulpotomies	tooth-list	List of teeth with pulpotomies done at first sedation

**Table 2:** Scores achieved by the best classifier found

Second sedation	Precision	Recall	f1
No	0.87	0.96	0.92
Yes	0.92	0.75	0.83

**Table 3:** Scores achieved by the best classifier found when conditioned on the Healthy variable for both classes [0 (absence), 1 (presence)]

Group	Size	Second sedation	sed/group	sed/total	Precision	Recall	f1
SHCN	121	48	0.39	0.2	[0.85, 0.84]	[0.9, 0.77]	[0.88, 0.8]
Healthy	109	7	0.06	0.03	[0.96, 1]	[1, 0.43]	[0.98, 0.6 ]

In summary, our Feature Selection algorithm is able to find a good set of predictors and returns an accurate predictor of the target variable. Let us remark that the size of the dataset is quite small, with only 230 records. Once a potential model has been found, a larger dataset might be compiled and further optimizations and refinements of the model may also be tested before being used in production.

### Analysis of features for inference

In this subsection we analyze the relevance assigned by the model to the different features with the help of SHAP explanations. All the figures shown have been taken from the report generated and displayed by our application. The inferences that can be extracted from this information are briefly commented but a further discussion and comparison with our results in the previous traditional study [15, 16], as illustrated in [14], is deferred to the Discussion section.

As general remarks, first let us note that the fact that the algorithm has chosen these particular 8 predictors indicates that they are more relevant than the other potential features, at least for prediction. Second, we use SHAP values for interpretation [11]. Briefly, a SHAP value computes the contribution of each feature to an individual prediction. That is, the SHAP value of a feature provides a numerical additive value to the final model output for an individual. However, one has to be careful regarding the quantitative aspects of the interpretation. For example, if the model predicts a probability of 0.8 for an individual to undergo a second sedation, and the *Healthy* feature is assigned a SHAP value of 0.16, to directly assign 0.16 of that total probability to being healthy or not is not meaningful, and may even be misleading [23]. Therefore, we will mainly discuss qualitative aspects, unless a quantitative interpretation is clear: SHAP values should be interpreted as the relative influence of a predictor in the final outcome, with the sign indicating the direction of the influence in the outcome, that is, a positive value indicates higher probability of second sedation, while a negative one indicates lower probability. Let us note also that quantitative claims are not common in classical studies either, where typically only acceptance or rejection of a null hypothesis with a measure of confidence is given, reported as “significant differences were found between the two variables”.

**Fig. 5:**
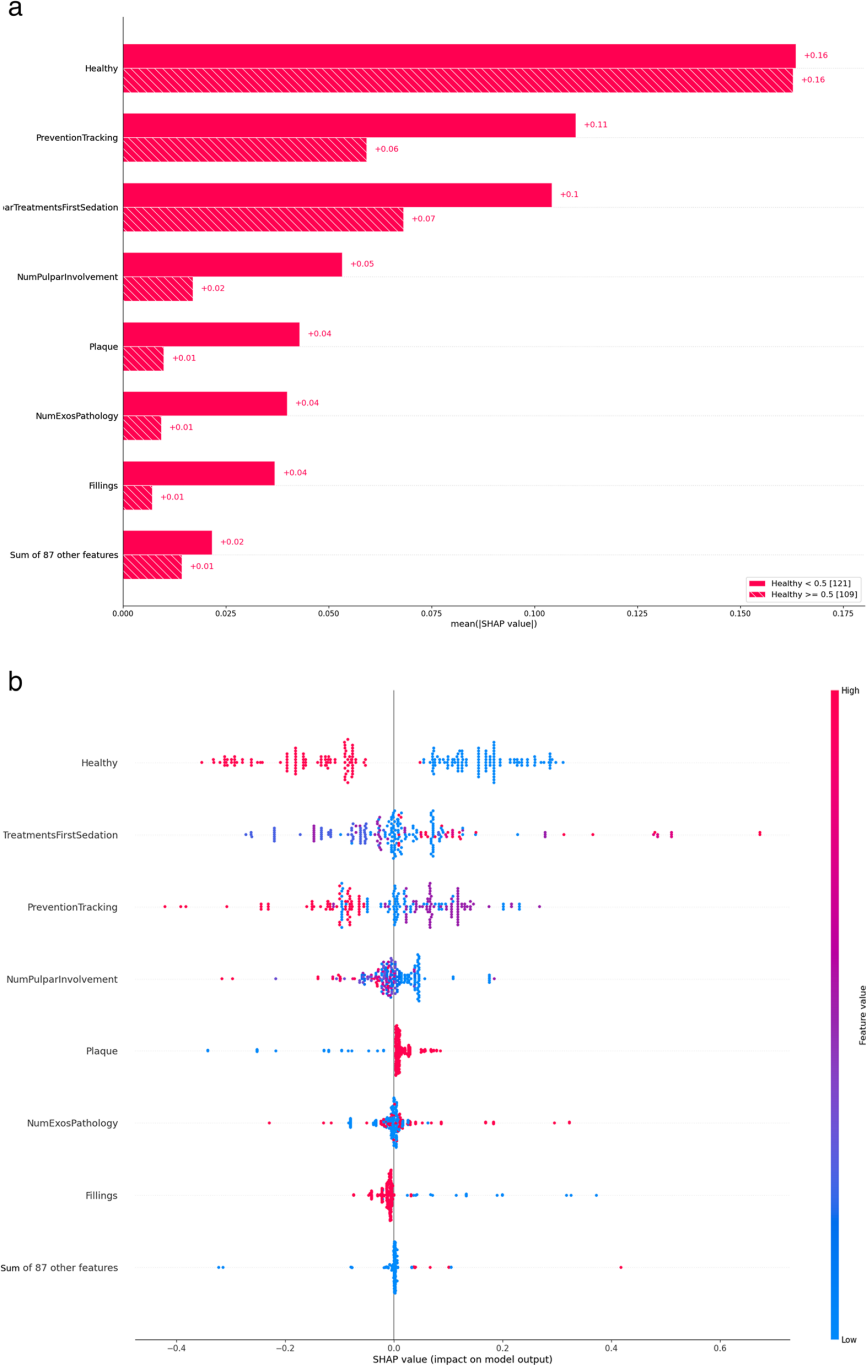
Feature relevance and impact on model output

In Fig. 5 we plot two views of the features’ relevance, according to their mean SHAP value. On the left, in Fig. 5a the mean absolute average value for each feature is plotted, grouped by healthy or SHCN patients. With this plot we can see the relative influence of each feature in the model output (the probability that a patient undergoes a second sedation) in absolute value, that is, without assessing whether the influence is positive or negative. While on the right, in Fig. 5b we show a scatter plot, which does show the direction of the influence as a function of the feature value. The value of the feature at each row is represented as a color scale, with red for high values and blue for low values. The assigned color depends on the type of variable, for instance, for a binary value, 1 is shown in red while 0 is shown in blue. For an integer variable, red represents the maximum, blue the minimum and purple hues values in between. This plot allows us to clearly see the influence of the different features in the final outcome. For instance, we see that being healthy clearly decreases the value of the output, and since that value is a probability, we see that it makes it less likely to undergo a second sedation.

As seen, the healthy status has the major influence in absolute value, as expected, since SHCN patients require more often a second sedation. Since the results are grouped by this feature in Fig. 5a, its value is equal for both groups. In Fig. 5b we can see the direction of the influence with the sign of the feature: healthy children (red points) SHAP values are negative, that is, tend to decrease the probability of a second sedation, while SHCN children (blue points) are positive and so tend to increase the probability of a second sedation. For SHCN patients, next in importance is the compliance with the prevention tracking program (*PreventionTracking* in Fig. 5a). However, for healthy children, the second more relevant feature is the number of teeth that required pulpar treatment at the first sedation (*NumPulparTreatmentsFirstSedation* in Fig. 5a). The number of teeth that showed pulpar involvement is the fourth more relevant predictive factor for a second sedation for both groups (Fig. 5a). Figure 5 also shows that the probability of a second sedation increases with the presence of bacterial plaque and a high number of extractions due to pathology during the first sedation.

**Fig. 6:**
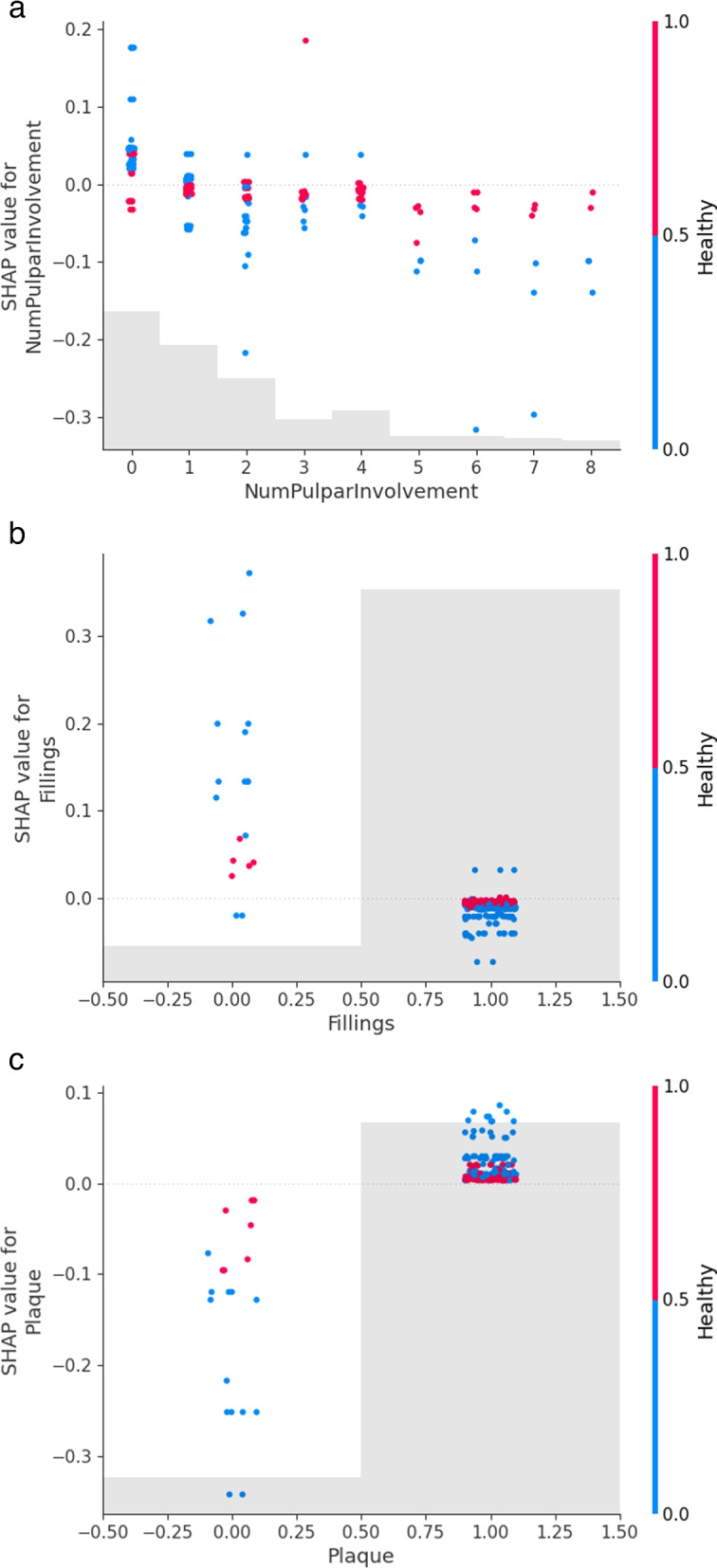
Dependence plot with interactions between variables. Brownish shaded bands show the histogram of the data in the X-axis. Interaction with the healthy status is displayed by showing healthy patients in red

Next, we turn to interactions between features. In Fig. 6 we plot the strongest interactions found by the model as dependence plots. These plots show the dependence of the SHAP value (Y axis right) with the value of the feature (X axis), and the interaction is depicted by coloring the points according to the value of another feature which has the strongest interaction with the former (Y axis left). The feature that has the strongest interaction is automatically computed by the application, but the user can also select other feature interactions that she might be interested in. As expected, most of the features have the strongest interaction with the healthy condition.

Figure 6a shows that, for SHCN patients, a higher number of teeth with pulpar involvement is associated with less likelihood of a second sedation. From Fig. 6b we see that the majority of the patients have undergone fillings during the first sedation, but it is noticeable that patients, especially SHCN, that did not receive that treatment are more likely to be sedated again. Finally, the model shows in Fig. 6c that the absence of plaque before the first sedation is a clear indicator that the probability of a subsequent sedation is low.

## Discussion

We separate our discussion into two parts: first, we focus on the technical aspects of our tool and compare them with previous similar works, then we discuss the relevant findings of our case study.

### Technical aspects

A general overview of ML methods in healthcare can be found in [2]. Their potential advantages are outlined in [1], while [7] discusses how to develop effective ML models for healthcare and related challenges and problems. As in other healthcare fields, the use of data-driven methods in odontology is growing quickly. Several systematic reviews focused in particular fields are available: Revilla-Leon reviews the application of ML methods in restorative dentistry [24] and also for gingivitis and periodontal disease [25]. More general reviews can be found in [26] and [27]. Most of the reviewed works are focused on diagnosis and prediction and in the majority of cases the ML methods have been applied to radiography and medical images. Particular and general systematic reviews stress the potential and accuracy of these methods but also warn that they are still in development.

We do not further discuss image-based methods, the technique most applied, but focus on the works most similar to our own in this paper, that is, those using clinical, behavioral, demographic, and laboratory data as input predictors for a ML model. A prominent example is given by Karhade et al. [28] who developed an automated ML model for classification of early childhood caries (ECC). As in our work, they performed a feature selection procedure, introducing different sets of plausible predictors, from a total number of 14 features, to the Google AutoML framework and performing an iteratively search process using the classification accuracy of the model for selecting the best performing model and proceeding with internal validation. But the feature selection procedure is not described in detail and no systematic search for features has been done. On the contrary, our feature selection algorithm tests all the combinations for a comparable size of the total set of predictors, or otherwise systematically searches the best combination for larger feature sets. In addition, ORIENTATE provides different global and individual interpretation plots to assess the relevance the model assigns to each predictor and support further inferences. Qu et al. [29] provide a prediction model for early childhood caries risk based on behavioral data. For feature selection, they manually remove the low-variance features and then use all of them to train only three different classifiers. They only provide the relative weights assigned by the model to the features used, without further explanatory reports. Campo et al. [30] evaluate different classifiers for the need for a root canal retreatment for a dataset with 205 cases, and propose a case-based reasoning algorithm with Bayesian networks that outperforms other types of classifiers. Unlike our work, they do not carry out a feature selection search but use all the features, and they extract the more relevant variables but do not provide further interpretability analysis or plots. Similarly, Thanathornwong [31] develops a Bayesian network decision support system for the prediction of the need of an orthodontic treatment. No feature selection search is done but they provide a graphical interface for the application and the results are compared to the assessments of two experts. Finally, Cui et al. [32] also trained several multiclass classifiers for tooth extraction, retention or restorative treatment. The authors do perform a feature selection algorithm after performing a feature extraction phase that recovers features and values from clinical records, retaining 34 features for the model. The most relevant features for the model are shown but no further explanation, interaction or interpretation plot is provided. Results were compared to expert predictions and showed that the model outperforms the experts.

Regarding the predictive capabilities of our models, our results agree with others [28], as expected, in that the number of features used as predictors is not directly correlated with the accuracy of predictions, see Fig. 4, and moreover, it is not obvious which ones are going yield the best-performing classifier. In Karhade et al. [28], they found that a parsimonious model including only two predictors (children age and the oral health status reported by parents) achieved the highest accuracy as predictors of ECC. A notable difference with this work is that they do not systematically search for the best combination of predictors, even though the size of their predictor set makes it computationally feasible (14 predictors). We believe the reason is that even though they use a reasonably user-friendly platform (Google AutoML), it still requires a certain technical background to truly automate a feature selection process, a problem that we intend to ameliorate with ORIENTATE. The accuracy of our best model is reasonably good (0.92 AUC, 0.75 recall and 0,92 precision) and higher than theirs (0.74 AUC, 0.67 recall and 0.64 precision), even though the size of their dataset is much larger (6404 samples) than ours (230 samples), which may also be due to their selected predictors. Therefore, we consider that an effective feature selection algorithm is essential for this kind of application and intend to improve ours as a future work.

Supporting feature relevance and global interpretation methods allows us to use our tool for statistical inference that can replace and/or complement classical statistical studies, as discussed by Bzdok et al. [14], where the similarities and differences between ML and classical statistical studies are analyzed. To elaborate on this point let us note that our application provides three main services: (1) to obtain a descriptive view of the dataset, (2) to find the best classifier model for a fixed set of features of interest (CE) and (3) to find the best subset of features and associated model from a broader set of features of interest (FS). The latter service is the most interesting from our point of view, because it allows to achieve two goals: first, it covers partially the goals of statistical inference, that is, it *draws inferences* about a population from the sample, which is the subject of what we call *traditional* studies. And second, it finds the combination of features and model that best *predicts* our target variable, which is the goal of ML algorithms typically. Let us clarify that inferences drawn by ML methods are derived by observing the subset of features used by the model *and* the contribution of each feature to the outcome (feature relevance) [14]. As an illustrative example, in our use case study our target is to predict the value of the variable *SecondSedation*, that is, the need to perform a second intervention under sedation for the subject, and we see that the best classifier uses, among others, the *Healthy* feature, that is, the absence of pathologies in the subject, and it is given a high relevance by the model. The equivalent procedure in a traditional study is to perform a statistical test against the null hypothesis that *Healthy* is associated with *SecondSedation* [14].

The ML approach to inference has some advantages: it can handle a large number of variables (features) and few samples, multiple testing and nonparametric models [14]. But it has several particular drawbacks too: ML models tend to use regularization to obtain the simplest model that predicts well, which means that it selects features that may capture efficiently the effects of other variables through correlations but are not meaningful to the user. For instance, in our case test, we find that the pulpar treatments on a particular tooth (75) have relevance for the prediction of the target variable, but which the actual relationship may be is not obvious at all. Additionally, ML models typically have worked as *black boxes*, where the user inputs some data and gets a result, without any clue about the actual process to reach the output [33]. Fortunately, a number of methods to explain ML output have been developed [33] and, in particular, the SHAP reports used here supply both individual and global explanations in intuitive formats, even though they have their own issues [23]. Both ML and classical statistic studies are complementary in several aspects: with FS, the ML approach can highlight relevant features quickly but is not appropriate in this form to examine the associations of particular variables that might be of interest to the practitioner but have been discarded by the model during the feature search. Instead, using CE is useful to test those cases, by including the variable of interest and examining the model results, which can and should be complemented by additional classical statistical tests.

As a summary, from the technical point of view, therefore, the distinguishing aspects of our proposal compared to previous works are: most of them use a custom application tailored for a specific target, while we provide a general purpose classifier evaluation application, and our tool supports systematic feature selection search and extensive interpretation functionality with a user interface designed for users with non-technical background. This latter aspect also allows ORIENTATE to be used to draw inferences about the features like classical statistical studies do. A limitation of our study is the small sample size of the dataset that we have used to test the application. Most of ML algorithms improve their performance with larger datasets. But, an advantage of our tool is that it is agnostic with respect to the dataset as we said, so it can be applied seamlessly to any other dataset in the proper format. As future steps we intend to collect additional datasets for evaluation. We should finally note that the interpretation of the features relevance may also be considered another limitation of our tool: the major risk of using predictive methods for inference is that incorrect interpretation of the model results in unwarranted causal inferences [34]. This kind of limitation is also acknowledged for example in [29], where they warn that some of the predictors were not considered causative. But this is a problem shared with classical statistical methods [34]. To tackle this problem, we plan to extend the functionality of ORIENTATE to apply causal inference methods to our datasets [34]: there are programming libraries [35] that can be seamlessly integrated with ORIENTATE.

### Case study

According to the relevance assigned to each feature by our model, the predictive factors were, in order, for healthy children: systemic health status, number of teeth with pulpar treatment at the first sedation, prevention tracking, number of teeth with pulpar involvement before the first sedation, presence of bacterial plaque, number of teeth extracted due to pathology and the number of fillings (Fig. 5a). For SHCN children, they were: systemic health status, prevention tracking, number of teeth with pulpar treatment at the first sedation, number of teeth with pulpar involvement before the first sedation, presence of bacterial plaque, number of teeth extracted due to pathology and the number of fillings (Fig. 5a).

According to our model, thus, healthy children would not need a second sedation, unlike SHCN children (Fig. 5b). This difference between both groups of children has also been observed in other published studies [36, 37]. For SHCN, complying with the prevention tracking program after the first sedation is a predictive factor for a second sedation. One of the goals of the prevention tracking program is to achieve a collaborative attitude in the patient, which would prevent future treatments under sedation. Thus, several authors believe that the lack of preventive programs or a bad design of them may be the cause of the reinterventions [36, 38–41]. However, few works have evaluated the effect of those programs in the long term [36, 42, 43]. In our previous study [15, 16] we showed that 80% of healthy children that complied with the prevention tracking program after a first sedation were able to receive at the dental chair treatments of relative complexity. On the contrary, due to their physical or mental limitations, only 18.4% of SHCN children were able to receive this kind of treatment at the dental chair. Despite it may seem contradictory, the fact that children show up at the prevention follows-up is a predictive factor for a second sedation. The reason is, as we said, that the prevention tracking program for this children, in addition to implement preventive measures, allows to carry out an early diagnosis of the therapeutic needs that will have to be implemented under sedation for them. A limitation here is that we have patients for whom the compliance of the prevention program has not been recorded. Let us recall that compliance with the prevention tracking program is a ternary variable. That is, some patients were referred from other clinics and returned after the first sedation, so their compliance with the prevention tracking program was not recorded. For those patients, the *PreventionTracking* feature takes value 2 and is shown in red in Fig. 5b, for children that did have complied with the prevention program the value is 1 and is shown in purple, while children that did not follow the program, with value 0, are shown in blue. We see that not-recorded values (red) decrease the likelihood of a second sedation, since those patients do not come back to the clinic in many cases and we do not have records about the evolution of their oral pathology.

For healthy children, the second predictive factor in importance according to ORIENTATE was the number of teeth with pulpar treatment at the first sedation. However, in our previous study [15, 16] neither the initial oral pathology nor the treatments done during the first sedation were indicators that could predict the need for a second sedation. But other works [36, 43] did report that children that received more conservative restoration treatment during the first intervention tend to need more retreatments under deep sedation.

The fourth predictive factor for both groups of children was the number of teeth with pulpar involvement before the first sedation. In fact, when the number of such teeth increases, the probability a second sedation decreases (Fig. 5b). In SHCN patients, a higher number of teeth with pulpar involvement is associated with less likelihood of a second sedation, and similarly for healthy children but in a less pronounced way (Fig. 6a). As we already found in our previous study [15, 16], SHCN children are usually older so most of those teeth with pulpar involvement were about to exfoliate. In those cases, the selected treatment was extraction at the sedation. This way, since those children have more extractions and, since due to having fewer teeth, the likelihood of dental pathology is lower, ORIENTATE decreases also the probability of a second sedation. This particular result allows us to remark that, to make inferences, we have to complement the model results and explanations with the rest of the data available, in this case, the age of the patients, a feature that was not selected by the FS algorithm.

The model also shows that SHCN children that have not had fillings done at the first sedation are more likely to be sedated again (Fig. 6b) and the absence of plaque before the first sedation is a clear indicator that the probability of a subsequent sedation is low and vice versa (Fig. 6c). This seeming contradiction is due to the fact that many SHCN children without caries lesions are sedated only to remove the large amount of tartar that they produce, due to their diet and poor hygiene, since they are not able to collaborate with the dentist for its removal. So, in our previous study [15, 16], we found that 3 out of a total of 7 healthy children (42,85%) needed a second sedation to undergo a tartar removal procedure, whereas 42 out of a total of 48 of SHCN children (87,5%) needed it.

In summary, we have shown that the predictive factors for a second sedation found by ORIENTATE are coherent and complement the ones previously found in our classical study. For SHCN children, the systemic health status was the more relevant, followed by prevention tracking, number of teeth with pulpar treatment at the first sedation, number of teeth with pulpar involvement before the first sedation, presence of bacterial plaque, number of teeth extracted due to pathology and the number of fillings. In addition, it is capable of detecting predictive factors, which would escape the usual analysis of clinical practice, and even may seem contradictory but, upon careful reflection, turn out to be consistent as we have discussed.

## Conclusion

ORIENTATE allows the automated application of machine learning classification algorithms on general datasets by clinical practitioners lacking technical skills. It can find the best subset of predictors for a given target variable and shows several graphs that facilitate the explanation of the classification model results, using global interpretation methods, and an interface for the prediction of new input samples. For the case study, our tool was able to achieve a high prediction accuracy for a second sedation in SHCN children, despite the dataset being heavily imbalanced and its small size. The analysis of the relevance of the features showed that, for healthy children, the number of teeth with pulpar treatments at the first sedation is a predictive factor for a second sedation, whereas for SHCN children a predictive factor was the compliance with the prevention tracking program. Our analysis is complementary to others done with classical statistical methods. In summary, ORIENTATE achieves several goals: first, it automatically finds suitable features and generates accurate classifiers for predictive tasks. Second, it helps researchers without specific skills in data methods in both the application of machine learning classification and as a complement to classical studies for inferential analysis of features.

## Data Availability

The source code of ORIENTATE as well as the datasets generated and/or analysed during the current study are available via the following link: https://gitlab.com/esteban.egea/orientate. Project name: ORIENTATE Project home page: https://gitlab.com/esteban.egea/orientate Operating system(s): Platform independent Programming language: Python Other requirements: python v3.9.12, flask v2.0.3, scikit-learn v1.1.1, shap v0.41.0, flask-cache v0.13.0.1, bokeh v2.4.3, xgboost v1.6.1, graphviz v0.20. License: MIT

## Abbreviations

AUC: Area Under the Curve
CE: Classifier Evaluation
FS: Feature Selection
ML: Machine Learning
ORIENTATE: applicatiOn of machine leaRning for classIfication of dENTal pATiEnts
ROC: Receiver Operating Characteristic
SHCN: Special Health Care Needs
